# Exploring Physical Activity Among Mexican American Immigrants in New York City Before and During the COVID-19 Pandemic: A Two-Wave Panel, Mixed-Method Analysis

**DOI:** 10.1007/s40615-024-02244-1

**Published:** 2024-12-05

**Authors:** Kathryn P. Derose, Neil Hwang, Sandra Verdaguer, María Hernández, Alyshia Gálvez, Aisha King, Ivonne Quiroz, Karen R. Flórez

**Affiliations:** 1 University of Massachusetts Amherst, 715 N. Pleasant St, Amherst, MA 01003 USA; 2 RAND, Santa Monica, CA USA; 3 Bronx Community College, City University of New York, New York, NY USA; 4 School of Public Health and Health Policy, City University of New York, New York, NY USA; 5 El Instituto: Institute of Latina/O, Caribbean, and Latin America Studies of College of Liberal Arts and Science, University of Connecticut, Storrs, CT USA; 6 Lehman College and the Graduate Center, City University of New York, New York, NY USA

**Keywords:** Latino adults, Physical activity, Social networks, Social support, COVID-19

## Abstract

The COVID-19 pandemic was associated with decreases in moderate-to-vigorous physical activity and increases in sedentary time for children and adults, and there is some evidence that Latino populations were particularly affected. This article leverages a two-wave panel, mixed-method social network dataset collected before and during the pandemic with Mexican American immigrants living in New York City to examine social networks and other factors associated with physical activity and explore participants’ perceptions about how the pandemic affected their physical activity. Participants (*n* = 49) completed in-person, egocentric social network interviews between January and June 2019 and virtual follow-up egocentric social network interviews between May and November 2021. Qualitative data collection with a subsample (*n* = 25) occurred between October and December 2022. Social network quantitative analyses found that age and female sex were negatively associated with participants’ baseline physical activity level, but the proportion of alters (important persons identified by the participant) with whom the participant did physical activity at baseline was positively associated. Baseline physical activity level was inversely related to the change in physical activity, and the change in the proportion of alters who were close to the participant was negatively associated with physical activity change at follow-up. Qualitative results supported the quantitative findings, providing rich narratives regarding the importance of social support for physical activity during the pandemic. Social networks among Mexican American immigrants in New York City provided motivation and support for physical activity but became harder to sustain during a pandemic. The findings can inform strategies to create environments that are conducive to physical activity while also protecting public health.

**Supplementary Information:**

The online version contains supplementary material available at 10.1007/s40615-024-02244-1.

## Introduction

The COVID-19 pandemic caused significant disruption in people’s lives globally. Beyond early “shelter-in-place” and “stay-at-home” orders issued by United States (U.S.) state governments in March and April 2020 and “lockdowns” in other countries, many businesses, schools, and other public and private spaces were closed for months. These closures had profound consequences on household income, physical and mental health, and health behaviors such as physical activity (PA) that contribute to longer-term health effects [1, 2]. Understanding the impacts that COVID-19 had on PA is important given that engaging in regular physical activity (PA) contributes to many positive health outcomes, including longevity; improved quality of life; reduced incidence of cardiovascular diseases, diabetes, depression, and certain cancers; and the prevention of obesity [3].

Systematic reviews of studies assessing the impact of COVID-19 on PA have consistently found decreases in moderate-to-vigorous physical activity (MVPA) for both children and adults [4–8]. Nevertheless, some studies report differential effects of COVID-19 on PA across gender and racial-ethnic groups. For example, studies in Spain, Australia, and New Zealand found decreases in PA among men, whereas women had increases [9, 10]. In addition, these studies and others in Europe have found that individuals who were most active prior to March 2020 experienced the greatest decrease in PA, while individuals who were previously less active increased time spent on PA [9, 11]. Studies in the U.S. comparing pre- and post-COVID-19 PA found large reductions of MVPA, with greater effects among those previously active [12, 13]. Additionally, evidence from the initial stages of the pandemic suggested greater reductions in PA among Latino populations than among other racial-ethnic groups [14]. As the largest minoritized group in the U.S. comprising 62 million people in 2020 [15], Latino populations’ PA is important not only for overall health but because they are disproportionately affected by chronic diseases such as diabetes [16], for which PA plays an important role in prevention and management [17]. PA among people with diabetes has been negatively affected by the pandemic due to both governmental restrictions and fear of contracting COVID-19 [18].

Social networks have the potential to improve PA among Latino and other populations through several related but distinct pathways such as social support, access to resources, social engagement, and social norms. Most of the literature on PA focuses on the construct of social support, and most of these studies do not formally measure social networks (i.e., the structural features of social relationships) [19]. For example, a systematic review evaluating the effectiveness of PA interventions among general populations found that interventions solely focused on social support (i.e., there was no formal social network assessment) in community settings were effective, whereas evidence was insufficient for family-based social support interventions [20]. Another systematic review focusing on PA interventions with Latino populations found that social support (defined as having a friend who is supportive, knowing or seeing people exercise, or participating in a group exercise class) was a common component and increased PA [21]. A review of PA intervention studies among minoritized populations found that interventions that included social support and encouragement for behavior change from family and peers had greater success in improving PA over time than any other psychosocial factor [22]. Partner support for PA has been found to be related to PA among Mexican American women [23]. Similarly, having an exercise partner, receiving help with household duties, being “very much” satisfied with support, and naming > 2 supporters were found to be positively associated with leisure-time PA among Latina women [24]. In terms of social network studies, a study of Mexican American families (parent–child dyads) found that mutual PA encouragement between parents and children was strongly predictive of co-engagement in PA, although only 15% of parent–child dyads mutually encouraged each other [25]. A systematic review of social network influences on adults’ PA found that network characteristics such as size, homophily, composition, and exposure to PA were all associated with individual PA [26].

In sum, although research has confirmed that the COVID-19 pandemic’s effect on PA depended to some extent on pre-pandemic activity levels and other personal characteristics, the extent to which social networks might have been protective or not for Latino populations has not been explored. This mixed-method manuscript explores the PA experiences of a Mexican American adult sample living in New York City before and during the COVID-19 pandemic. Specifically, we leveraged a two-wave panel, mixed-method egocentric social network dataset that asked participants (termed egos) to identify their most important personal connections (termed alters) and the extent to which they provide (or fail to provide) support for PA. This approach allows for the computation of structural features of the network (e.g., size, density) while also allowing for the quantitative examination of the provision of social support for PA by network members from baseline, as well as the changes from pre- to post-COVID-19. Using qualitative data, we further explore the study participants’ perceptions about how the pandemic affected their PA. Specifically, we explore

1. How are social network factors associated with Mexican American adults’ PA before the pandemic? (using quantitative egocentric social network data pre-COVID-19)
2. How are these social network factors associated with the change in Mexican American adults’ PA during the pandemic? (using quantitative egocentric social network data pre- and post-COVID-19)
3. How do participants describe the factors affecting their PA during the pandemic? (using qualitative data collected post-COVID-19)

## Material and Methods

This study used an explanatory sequential mixed-method design, with quantitative data collection first, followed by qualitative data collection [27]. Participants were immigrant adults who self-identified as Mexican, lived in or near the Bronx, and were recruited through a Catholic church. Sampling and recruitment details have been described elsewhere [28]. Collection for the first wave of quantitative data occurred in person between January and June 2019, and the second wave of quantitative data collection occurred virtually in May–November 2021. For the quantitative data reported here, we include only participants who participated in both waves of data collection (*n* = 49). Qualitative data collection with a subsample of *n* = 25 occurred between October and December 2022. All data were collected in English or Spanish depending on participant preferences, by trained bilingual research assistants. Approval was granted by the Ethics Committee of the City University of New York and the RAND Human Subjects Protection Committee, and participants provided written consent.

### Quantitative Data Collection

This study took an egocentric approach to measuring personal social networks, where participants (“egos”) were asked to enumerate the 20 most important persons in their lives (called “alters”), describe how they know them, and indicate whether they had been in contact with them (face-to-face, by phone call, text/phone app message, email, or social media). During wave 1, the period of consideration was the past 6 months, while for wave 2, the timeframe was 12 months. Participants were then asked about their alters’ demographics, frequency of contact, relevant health characteristics, and, to determine if their personal network is densely or sparsely connected, the extent to which the alters knew each other. They were also asked about how close they were to each alter and whether they did PA with them as well as changes to these factors during COVID-19, as described below. Standard elicitation procedures for personal social networks were used [28]. Other data used in this study were collected by validated survey measures (see below), administered in person in 2019 and via Zoom/phone in 2021.

### Measures

#### Dependent Variables

**PA**: In both waves 1 and 2, participants’ PA was measured using the validated International Physical Activity Questionnaire (IPAQ) Short Form (IPAQ-SF) [29]. The IPAQ-SF asks individuals about PA in the past 7 days, including work-related PA, house and yard work, transportation PA, and leisure-time PA [29]. Individuals were asked 2 questions for each of 3 PA levels (vigorous, moderate, and walking): (1) the number of days the individual did at least 10 min at a time of this activity level and (2) the number of hours or minutes that the individual does at least 10 min of that level of PA on a given day. Results were used to compute PA levels using a standard algorithm [30]: 1 (low), 2 (moderate), and 3 (high). Wave 1 (pre-COVID-19) IPAQ level is the first dependent variable, and change in the IPAQ level (the difference between IPAQ levels in the two waves) is the second dependent variable.

#### Network-Level Independent Variables

**Percentage of alters close to the participant in wave 1; changes in the percentage of alters close to the participant across waves**: Participants were asked about their relationship with each person in their network (“alters”) to assess the degree of closeness. Responses were solicited on a four-point scale ranging from “strongly agree” to “strongly disagree.” Those indicating either “strongly agree” or “agree” were coded as network members perceived as close to the participant. We then computed the proportion of network members falling into this category out of a total of 20. To assess changes over time, the percentage change was calculated for each participant by subtracting the percentage in wave 1 from that in wave 2.

**Percentage of alters with whom the participant did PA in wave 1; changes in the percentage of alters the participant did PA with across wave**: These measures were assessed with the question “In the past 12 months, who did you do PA with (for example, walking, bike riding, sports, or exercise classes) on a regular basis (at least a few times a month or more).” Alters indicated in the responses were coded as network members whom the participant did PA with. We then calculated the proportion of network members (out of 20 total) who fell into this category. The change in this percentage was computed for each participant by subtracting the percentage in wave 1 from that in wave 2.

#### Individual-Level Independent Variables

**Sociodemographic**: Each participant was asked to report their sex, coded as either “female” or “male,” and their birth year, which was used to categorize individuals into one of the following age groups: 0–30, 31–40, 41–50, 51–60, and 61–100 years. In addition, participants were asked about marital status (single/never married, married, living with a partner, divorced, separated, or widowed) and work status (working full-time, working part-time, self-employed, unemployed and looking for work, retired, disabled, student, or homemaker; these were collapsed into working part-time or full-time or self-employed vs. others).

**Body mass index (BMI)**: BMI was objectively measured in wave 1. Specifically, interviewers were trained to measure height using a portable height measuring board by SECA. Height was recorded to the nearest one-eighth of an inch, and any adjustments were recorded (e.g., for shoes or hair ornaments that the respondent chose not to remove). Respondents’ weight was measured using the SECA Robusta 813 digital scale, which is capable of weighing respondents up to 400 lb. We took these objective measurements and applied the standard formula of weight (kg) divided by height squared (m^2^) to derive BMI.

**Number of children under the age of 16**: For each participant, this measure represented the number of alters younger than 16 years old.

**Baseline IPAQ level**: For the dependent variable “change in IPAQ level,” participants’ baseline IPAQ level, measured as their IPAQ level wave 1, was used as an independent variable.

#### Statistical Analyses

Descriptive statistics were computed for each wave, including changes between waves. Given the ordinal dependent variables, we used ordered logistic regression to assess the factors associated with participants’ PA at baseline (wave 1) and change in PA during COVID-19 (wave 2). We hypothesize a specific ordered logistic regression model called the proportional odds model (also known as the cumulative logic model), which allows each ordinal level a different intercept but assumes an identical slope at all outcome levels. We assessed the appropriateness of this parallel slope assumption of the proportional odds model by performing the Brandt test and found that the parallel line assumption holds for each model we discuss in this paper. For the cross-sectional analysis (baseline, wave 1 data only), the ordered logistic regression model could accommodate the full set of control variables (age, sex, BMI, marital status, work status, and number of children as alters). For the longitudinal analysis (change from wave 1 to wave 2), the model could accommodate fewer number of covariates; thus, we included age, sex, and BMI as these are consistently associated with PA [31]. As a measure of model fit, we provide the Nagelkerke/Cragg-Uhler pseudo-*R*^2^, as its value ranges between 0 and 1, allowing for a natural interpretation similar to the *R*^2^ in ordinary least squares regressions. Statistical significance was set at *p* < 0.05. Because of the small sample size and the exploratory nature of the study, we also considered a relaxed threshold of *p* < 0.10. All analyses were conducted using R version 4.3.1.

### Qualitative Data Collection

The qualitative sample at wave 2 (*n* = 25) was purposively sampled from the quantitative sample from wave 1, which is described elsewhere [32]. The entire sample was characterized via their connectivity at wave 1 (“low,” “moderate,” and “high”), and information-rich cases were sampled from each stratum [32]. Specifically, based on their quantitative social network data, participants were classified as having strong network connectivity if they were in the upper quartile of several network characteristics (i.e., top 25%), moderate connectivity (i.e., most characteristics in the mid 2 quartiles), or weak connectivity (i.e., most characteristics in lower quartile) [28]. Individuals who had participated in in-depth interviews at wave 1 were prioritized for interviews at wave 2. Of the 25 wave 1 interview participants, 13 were not available for the wave 2 interviews and were replaced with 13 other wave 2 participants, bringing the total wave 2 semi-structured interview sample to *n* = 25. Individuals received $50 gift cards for participating in the semi-structured interviews, which took place via Zoom (18 interviews), WhatsApp (1 interview), or in places of the interviewees’ choosing (6 interviews). The interviews were conducted in participants’ preferred language (Spanish or English) and lasted between 46 and 120 min. Interviews were audio-recorded with permission.

The semi-structured interview guide was made up of two overall sections, each containing between 6 and 7 questions and additional prompts to facilitate in-depth discussion of the topics within each section. The first section asked about their social networks using visualization data (e.g., their relationship with each alter, interactions during COVID-19, etc.). The second section asked about the extent to which specific alters provided information and support related to healthy eating and physical activity (and/or made it difficult to eat healthy foods or to be active) and general interactions with alters around these issues during the pandemic. This latter section had specific questions that probed about participants’ PA before and after COVID-19 and the reasons for these changes.

#### Coding and Analysis

Audio recordings were transcribed verbatim in Spanish or English via Sonix, verified by one of the interviewers, and uploaded in their original language to Dedoose, a qualitative data analysis software program that facilitates coding and data analysis (Dedoose Version 9.0.17, 2021). Coding procedures consisted of qualitative content analysis methods [33] using deductive approaches while also allowing for emergent codes or inductive approaches [34, 35]. A preliminary codebook was developed based on the interview guide, and as themes emerged, codes were either added, deleted, or merged. Three trained bilingual coders coded each transcript, meeting weekly to discuss codes that needed to be modified or adjusted, and the categories used for this analysis included the following: PA before COVID-19, PA during COVID-19, PA after COVID-19, social support for PA, and lack of social support for PA. Coded excerpts were transferred into an Excel matrix with each participant in a row and code categories across the columns to do a cross-participant comparison and identify primary themes for each code category. All English translations were done for this paper by the first author and were reviewed by other bilingual co-authors.

## Results

### Participant Characteristics

Table 1 provides summary statistics for individual- and network-level variables at waves 1 and 2. A majority (76%) of the sample was female, aged between 31 and 50, and had a median BMI of 29.3. Most were either married (37%) or living with a partner but not married (20%); 29% were single, and 14% were divorced, separated, or widowed. A little less than half (39%) were working (full-time, part-time, or self-employed). Between waves 1 and 2, there was an increase in moderate levels of PA (from 29% of participants falling in this category at wave 1 and 63% at wave 2) and a decrease in high levels of PA (from 26% of participants in this category in wave 1 to only 6% in wave 2). Regarding network-level characteristics, participants reported that they felt close to an average (median) of 95% of their network at both waves 1 and 2. Participants reported doing PA with a higher median percentage of their network at wave 2 (median 10%) than at wave 1 (median 5%).

**Table 1:** Summary statistics for individual-level and network-level variables at waves 1 and 2 (*n* = 49)

Individual-level variables (count)	Wave 1	Wave 2	Change
Physical activity level (IPAQ)			
Low	22 (45%)	15 (31%)	
Moderate	14 (29%)	31 (63%)	
High	13 (26%)	3 (6%)	
Change in IPAQ level			
− 2			4 (8%)
− 1			9 (18%)
0			22 (45%)
1			14 (29%)
Sex			
Male	12 (24%)		
Female	37 (76%)		
Age			
18–30	6 (12%)		
31–40	13 (27%)		
41–50	19 (39%)		
51–60	9 (18%)		
61–100	2 (4%)		
BMI			
Median (IQR^1^)	29.3 (21.8, 36.8)		
Mean ± SD^2^	29.9 ± 5.3		
Marital status			
Single, never married	14 (29%)		
Married	18 (37%)		
Living with partner, but not married	10 (20%)		
Divorced, separated, or widowed	7 (14%)		
Employment			
Working full-time, part-time, or self-employed	19 (39%)		
None of the above	30 (61%)		
Network-level variables (percentage in network)			
Alters the ego feels close to			
Median (IQR^1^)	95 (85, 100)	95 (80, 100)	0 (− 10, 10)
Mean ± SD^2^	88.4 ± 18.4	88.8 ± 17.2	0.5 ± 14
Alters the ego does physical activity with			
Median (IQR^1^)	5 (0, 25)	10 (0, 30)	0 (− 15, 15)
Mean ± SD^2^	12.7 ± 15.9	12.7 ± 14.7	0 ± 14

^1^Interquartile range

^2^Standard deviation

### Factors Associated with Participants’ PA Before COVID-19

Table 2 provides the proportional odds model regression results on the factors associated with participants’ PA during wave 1 or baseline (pre-COVID-19). We see that the proportion of alters who did PA with the participant at baseline was positively associated with participants’ PA at baseline, whereas age, female sex, and living with a partner (vs. being single) were negatively associated.

**Table 2:** Factors associated with participants’ baseline (wave 1) IPAQ levels (*n* = 49)

	Coef	SE^1^	*P*
Age	− 0.155	0.093	**0.095**
Female sex/gender	− 2.908	1.676	**0.083**
Proportion of alters who are “close”	− 0.017	0.039	0.662
Proportion of alters who do PA with participant	0.057	0.026	**0.032**
BMI	− 0.074	0.093	0.427
Marital status			
Single	REF	REF	REF
Married	− 1.795	1.571	0.253
Living with partner	− 4.342	2.125	**0.041**
Divorced, separated, or windowed	− 0.213	1.941	0.913
Working^1^	− 0.013	1.205	0.991
Number of children < 16 yrs old as alters	− 0.131	0.773	0.866

Pseudo-R^2^ = 0.92; ^1^﻿Working full-time, part-time, or self-employed vs. all others

### Factors Associated with Change in Participants’ PA Pre- to Post-COVID-19

Table 3 presents the proportional odds regression output for the change in IPAQ scores. Wave 1 IPAQ level has a significant negative association with the change in IPAQ levels, demonstrating that those who engaged in higher (lower) physical activity in wave 1 subsequently decreased (increased) the level of physical activity in wave 2. In addition, an increase in the proportion of alters who were considered “close” across waves was associated with a reduction in IPAQ levels at wave 2.

**Table 3:** Factors associated with a change in participants’ IPAQ level from wave 1 to wave 2 (*n* = 49)

	Coef	SE	P
Baseline IPAQ	− 4.103	0.981	**0.000**
Age	0.025	0.035	0.464
Female sex	− 1.478	0.886	**0.095**
Change in proportion of alters “close”	− 0.066	0.030	**0.026**
Change in proportion of alters do PA with	− 0.009	0.025	0.719
BMI	− 0.024	0.068	0.720

Pseudo-*R*^2^ = 0.74

### Qualitative Themes

Analysis of the qualitative data identified three broad themes about how COVID-19 affected participants’ PA: (1) how COVID-19 constrained PA, (2) how the pandemic enabled some to become more active, and (3) the importance of social support from family and friends that motivated people to remain physically active during the pandemic. We review in detail each of these three themes below, providing illustrative quotations from participants.

### How COVID-19 Constrained PA

A common overall theme among some participants was how the pandemic constrained their ability to do PA and led to a decrease in PA. There were several reasons or explanations given for how COVID-19 constrained or curtailed their PA. These included perceptions that they should avoid leaving their homes during the pandemic, that specific places where they liked to do PA such as workplaces (during breaks) and local parks were closed, and that social distancing meant they could not exercise with others and all interactions needed to be virtual. Women with young children reported the largest challenges to PA.

Participants’ perceptions that they should stay home stemmed from concerns regarding the pandemic itself as well as local laws and restrictions on gathering in public spaces. For example, one woman stated, “I didn’t even think about [going out], because I knew that we had to be shut in at home” [50-year-old married woman]. Similarly, another woman expressed fear about going out: “But how are we going to go out if we didn’t know….we were afraid to go out…we would look out the open window and sometimes, nobody, nobody, nobody, nobody, nobody was in the street” [53-year-old woman living with a partner]. Some participants, particularly young men, emphasized that their reluctance to leave home was about “respecting the law” and avoiding trouble with the police, as described by this young man:

Well, during the pandemic I hardly did any [physical activity]. It was only cooking and sleeping…well, because we couldn’t go out…more to respect the laws in place about not going out to walk….I went out only to buy food, [then] returned to eat, cook. All that…I didn’t do [physical activity]… only watched TV….I couldn’t [go out]…I wanted to obey the law about not going out…so I wouldn’t have trouble with the law…to respect the law, but also because the police were always surveilling” [23-year-old single man].

Similarly, another man described how their usual soccer games could not happen because of COVID-19 and police intervention: “And we used to do that every day, Monday through Thursday. We used to play soccer before COVID. But, you know, when COVID hit, we couldn’t do anything…if we went to play and someone called the cops on us. And then [the police] would come” [30-year-old single man]. Another man described how he used to play soccer at work during breaks but that was lost during COVID-19: “[We used to play] soccer…at work, when we had a break, we would start playing….we had an hour and a half, [so we played during our breaks], one hour and later a half hour” [27-year-old single man].

Parks were a common place where both women and men participants described doing PA with family and friends before the pandemic. At the onset of the pandemic, individuals felt that they could no longer visit parks. One woman said that even the parks were closed during the pandemic: “But, look at the coincidence, that with the pandemic, they closed the parks…they weren’t open. It’s been like a month since they opened up here. [Only] two parks now” [48-year-old single woman].

Several participants expressed that exercising with others was more difficult during the pandemic because of the need for social distancing. In response to a question about whether there were people who encouraged her to exercise during the pandemic, one woman shared, “Really, no, because of what I said [before], we had to be socially distanced. No, no, not that. Well, even if we said, ‘Let’s go running, let’s go, yes!’ We said we were going to go run [together]…but nothing ever happened” [45-year-old married woman]. Another woman explained that she is normally very active and had started to become active again, but during the pandemic, everything had to be done via Zoom:

Now I’m becoming more active. I like walking a lot. Almost every day I am doing something, I have a lot of activities, I look for a lot of things to do, My days are very busy. Thus, I am becoming more like [before], when I liked to go out, do things. But during the pandemic, yes, I stopped doing these things, no, I didn’t do hardly anything, only via zoom, everything by zoom [35-year-old woman, living with a partner].

Women who had babies during COVID-19 described more challenges to doing PA:

Yes, [I used to do] biking and running. Yes, much has changed, more than anything, two things contributed: the fact that I became a mom and the pandemic. Thus, one thing and the other came together and as I said, now I feel [too] tired to give that “extra” [34-year-old living with a partner].

How did [my exercise routine] change? Well, for the worse, because no, no, no, there hadn’t been much movement and in that time I was pregnant, thus, there wasn’t much movement, we could say. I tried to move myself, but, no, not at 100% [38-year-old woman living with a partner].

### How the Pandemic Enabled Some to be More Physically Active

Although many participants described how COVID-19 constrained their ability to do PA, there were some participants who indicated that they became more active during the pandemic. The explanations for this included that the pandemic shutdown gave them more time at home and thus more time to do things they considered healthy (e.g., PA; healthier, home-cooked meals), worrying about weight gain, avoiding boredom-induced unhealthy behaviors (e.g., smoking), and staving off negative feelings like depression.

Participants described how working from home gave them more time to dedicate to living a “healthy lifestyle,” as described by this man:

Well, before [COVID] it was a little complicated because I had a job that was more demanding. Back then, I left at 7 in the morning and came back sometimes at 10 at night. You would get home dead and you didn’t want to do anything. And sometimes on Saturdays and Sundays I had to be away for work. So, when the pandemic came everything changed and we started having a little healthier lifestyle of eating at home [and] doing exercise, even if behind closed doors. And actually, it has changed a little, since we’ve gone back to work, you don’t have the time sometimes to go back to do what you used to do [45-year-old married man].

Similarly, a woman described her healthier routine during the pandemic:

Ah, yes, the routine [changed] a lot because you know, you stop doing certain things and you do other things. I started doing more exercise….and stopped eating so much, instead I ate less and did more exercise and ate more salads [41-year-old woman, married].

One woman described getting trained as a Zumba instructor with her increased spare time:

Oh, I did more [exercise]….because I tried to eat well and do exercise…I had more time alone, so I started doing my exercises. I even paid to go get trained as a Zumba instructor because I had a lot of free time. Well, I was serving here, but because nothing else was happening, well, in the meantime, I dedicated myself to dancing [40-year-old woman, separated]

Another reason given by participants for their increased physical activity during COVID-19 was their concern about weight gain and other health issues. For example, this participant described being inspired by a friend’s weight loss: “Well, he was a little chubby, but now he is already losing weight. He does exercise so he doesn’t become desperate….He would say to us…’Let’s go [exercise]!’]” [27-year-old single man]. Several participants had chronic disease that compelled them to do exercise:

Yes, yes, [I felt the need to do exercise during the pandemic] because I didn’t do anything and also for my health, for me and my blood pressure, I need to do exercise. Right now, I don’t do a lot, but when I go sell, because from here I go to 154^th^ [and] I carry all my things there, to 3^rd^ Avenue. And there I do pretty well, because it is something good for my state of mind. I also share with people there and I have a good time and then from there I come back with my stuff until I get here [59-year-old woman living with a partner].

Participants who increased PA during the pandemic commonly cited walking as a primary means of activity: “Well, [your exercise routine] changes when your job changes, the daily life you have….During the pandemic what we did was walk and walk more. For example…we went out to walk daily…to keep your mind occupied, breathe, be active” [45-year-old woman, living with a partner].

Nevertheless, many participants talked about periods during the pandemic when there was fear of leaving home. In those times, some found other resources to do PA at home, like YouTube videos.

We went out to walk [with my wife] very little because at that time, one was afraid so that you didn’t want to go out. She would look for many videos in YouTube and there are influencers around that did exercises at home, from jumping jacks, sit-ups, pushups. Thus, occasionally I would [exercise] with her in that way….I did more walking, because I was the one who went out to do errands, to do some things. She stayed home. But most of the time, it was at home and those types of exercises. Walking, well, obviously, when I came back, sometimes we would go out to walk the two of us, at least twice a week, together. But in terms of exercising at home, yeah, that was most days [45-year-old married man].

Participants described exercising at home both for health reasons and to avoid falling into unhealthy habits and depression. For example, one man describes how exercise helped distract him from smoking: “[I did more exercises because of] boredom, to distract myself a little….from the people I was with because I felt that if I stayed there, I was going to start smoking or stuff like that, like it was going to make me need to smoke more. So, I just went out to walk” [23-year-old single man].

### The Importance of Social Support for PA by Personal Network Members

A final theme that emerged from participants’ narratives about their PA during the pandemic was related to the importance of social support, especially for women participants. Much of the support that individuals received came from family and friends and involved encouragement, role modeling, doing PA together, and, in some cases, providing economic support for PA (e.g., gym membership).

Participants described how encouragement from their spouse, family, and friends was critical in getting them to do PA. For example, this man explains how he did not see the need for PA, but his wife did, so they did it together:

Well, I didn’t feel so much need [to do PA], but sometimes your partner makes you, right? And then you start doing it. Because the truth is that suddenly you were resting a little more. I mean, who doesn’t like resting, right? Getting up a little later and watching a little more television, etc., but needing to do [physical activity], no. But obviously she did see the necessity [of doing physical activity] and she transmitted it to me, so then both of us started doing [physical activity] [45-year-old married man].

Similarly, this woman described that when she lacked motivation to do anything, her sister encouraged her:

Well, sometimes, I’m being honest….there were days….I didn’t go out and wasn’t very excited about [going out], but I would call one of my sisters, and she would say to me, “Ah, no. Go shower so you get rid of this laziness.” And this is how she encouraged me. Or I started doing something or if I saw something, because it’s also like this, that sometimes the people who love you encourage you, right?[43-year-old woman, living with a partner].

Participants also talked about how their friends’ examples motivated them, for example, a woman described being influenced by her friends’ exercise and eating habits:

Because I used to visit them a lot, and I saw how they had their [exercise] machines, they would do exercise, and their refrigerator, when I visited them, I saw how they didn’t eat certain things. [This was] before and after COVID that they did this. Today, it’s another friend [named XX] who has pushed me that way also [41-year-old married woman].

The most frequent way that participants described how social support helped them do more PA was with family members and friends doing PA with them. This was particularly the case among women participants. These women described various examples of how they did exercise with their spouses:

Before the pandemic, I think we went out more often. Because of the pandemic we stopped going out because we had to wear masks and it was really very uncomfortable. If before [the pandemic] we went once a week, then it was every 2 weeks [during the pandemic]. [My partner] had some weights so he would do exercise here. He would also have our little boy walk on the stair exerciser that we have and do a little exercise. That was what [helped him] lose [weight] [50-year-old woman living with a partner].

[With my husband, we] exercised at home, like dancing, Zumba, cardio…sometimes 3–4 times [per week]….[watching videos] in YouTube….sometimes we went out [to parks] to run with my husband. But just sometimes, not a lot [34-year-old woman living with a partner]

Sometimes I go with my husband [to exercise] because my kids…[one of] my daughters….goes to work and the only one I have is [my other daughter] who is 19 years old, she goes to school, [then] to work. But I always do [exercise] with my husband who accompanies me [56-year-old married woman].

Women with young children talked about how they were able to exercise at home with their kids. For example, this woman described how her kids loved to dance:

Then my daughter, the younger one, says to me, “Mami, let’s dance.” She puts Netflix on the TV and starts to dance. Sometimes I join her, all my kids put videos on. This is doing exercise, but it’s fun and “mami let’s do it” and “mami, ok,” as long as they do, there [I am] their mami, there also doing with them the things they do on the TV. Somehow it motivates them and it’s good for me once in a while, that’s why you need to be with your kids, because they are our kids and if you don’t do it, who is going to do it, right?[36-year-old woman, living with a partner].

Women also described examples of how their women friends exercised with them.

[My friend said] that we should go running, do exercise, that we needed to move. Yes. She invited me to go out to run in the park [38-year-old woman living with a partner].

The truth is that during the pandemic, well, I didn’t feel like doing [physical] activity, I didn’t go out much. I started to go out little by little because [going out] made it difficult to breathe [after I had COVID]. I felt really tired, I couldn’t walk much. But little by little, I started going out to walk, “let’s go to the park,” and we go. [My friend] does yoga. The other day she invited me to her yoga class. I went with her to participate in the last yoga class that she had. And yes, she has always invited me, but I would say, “No, it’s still not time, a little later.” But I do go out to walk [54-year-old married woman].

Although participants mentioned friends inviting them to exercise, there were economic barriers that prevented them if said exercise was to occur at a gym, as explained by this woman: “I have another friend, she works at the community center, and she always asks, ‘When are we going to the gym?’ I like this idea, but I don’t know, I need to see how much the gym is going to charge” [45-year-old married woman]. These economic concerns highlighted the importance of joint social and tangible support from family and friends. For example, one woman explained that her husband purchased gym equipment for home: “Now I won’t have any excuses because my husband bought a Maxi Climber. And he is motivating me to lose weight because I got a little chubby. You know when we met, I was an extra small [clothes size] and now I am already in a large” [45-year-old married woman]. Another woman explained how one friend paid for her gym membership and another accompanied her and motivated her to exercise during times when there was no work: “Well, [friend1] paid for my gym [membership], and [friend2] motivated me so we would go exercise. [He motivated me] by accompanying me…because there was very little work as well, so we went to exercise” [59-year-old woman, living with a partner].

## Discussion

In this mixed-method, two-wave panel study, we explored how the COVID-19 pandemic affected PA among this sample of Mexican American immigrants in New York City. We found quantitative evidence that having a larger proportion of alters who did PA with the participant was significantly and positively associated with PA at baseline (pre-COVID-19). But post-COVID-19, we found that an increase in the proportion of alters who were “close” was associated with a reduction in PA. We also found quantitative evidence that baseline PA was inversely related to PA change post-COVID-19. Qualitative results largely supported the quantitative findings, demonstrating that whether the pandemic constrained or provided increased opportunities for PA varied across individuals due to their work and family situations. The qualitative data also provided rich narratives regarding the importance of social support for PA during the pandemic.

The baseline finding that the proportion of alters with whom participants did PA was positively and significantly associated with their baseline PA levels is aligned with previous findings. For example, a systematic review of social network studies among adults found that exposure to PA through network ties was one of the most salient network properties related to individual PA [26]. Similarly, in a study of personal networks among residents of low-income communities, a higher percentage of physically active network members was associated with higher odds of meeting PA guidelines [36]. Egocentric network data from Latino adults tend to suggest that larger network size is associated with leisure-time PA [37, 38]; other aspects of networks such as familial ties, contact frequency, and ego-alter dissimilarities in age (e.g., participants having younger children at home but also adult children) are also related to Latino adults’ PA [38]. However, in this previous study, examining various types of alter support available to Latina participants (informational, emotional, and instrumental including exercising with the ego), only co-participation in exercise was associated with both self-reported and objectively measured MVPA [38]. Our study found a positive relationship between the proportion of alters with whom the participant did PA and the participants’ PA levels—meaning that not only having someone to do PA with was important for PA level but also the number of people. This finding also demonstrates the contribution of measuring social networks when studying PA.

But in the context of a pandemic that restricted people’s mobility and ability to exercise with others, this facilitating factor of having more people to exercise with was not associated with PA change. Of note, baseline PA was a significant negative predictor of PA change, accounting for 61% of the variation in IPAQ change. In addition, we see that post-COVID-19, an increase in the proportion of alters who are “close” was associated with a reduction in participants’ PA levels. This could be a result of lower PA levels of close alters or increased caregiving responsibilities associated with these close alters. Future studies could examine the norms and beliefs of close alters and how this is related to the ego’s PA and other health behaviors.

Qualitative data helped explain some of the quantitative findings by highlighting how “lockdown” and social distancing messages discouraged participants from exercising with others and/or how places where they did PA such as workplaces (during breaks) and local parks were closed. Pre-COVID-19 research in Los Angeles found that urban parks were an important resource for PA and socialization among Latino immigrants [39], and another local study (in St. Louis) during COVID-19 found that Black, Latino, and young people were less likely to visit the park than others [40]; thus, policymakers must recognize the potentially disproportionately negative effects such closures have on certain populations and prioritize strategies to mitigate these effects. Some of our study’s participants said they became more active during the pandemic because they had less hectic lives and thus were able to do things they considered “healthy” such as PA and home-cooked meals, suggesting that addressing upstream factors like occupation and housing (e.g., necessitating long commute times) are essential to enable Latino populations and other marginalized groups to implement healthier behaviors. Social networks can help Latino adults overcome some of the barriers to PA through family and friends encouraging and modeling PA, doing PA together, and, in some cases, providing economic support for PA (e.g., gym membership), supporting theory and evidence that PA interventions that utilize and build on social networks can be particularly effective among Latino populations [22]. Further research is needed to determine how social networks can be harnessed to intervene on PA levels, above-and-beyond supportive network members and especially during a public health emergency like COVID-19 [41].

In terms of limitations, our sample was relatively small and recruited from one Catholic church, which may affect the power and generalizability of the quantitative analyses. Second, many of the measures were self-reported, including the primary quantitative outcomes (PA and change in PA), which may be subject to bias. Nevertheless, it is rare to have personal network data from immigrant populations at two time points before and during a pandemic and mixed-method data from the same population. Quantitative data from before COVID-19 and then collected again during the pandemic allowed us to examine changes in PA and social network factors that were associated with these changes. Exploring qualitative data that was collected during the pandemic and after the quantitative data made it possible for us to see whether these supported the quantitative findings but also provided depth and context for how the pandemic affected participants’ PA. Together, these mixed methods provided a more nuanced understanding of pandemic impacts on this vulnerable populations’ physical activity and well-being than would have been possible with only one method alone.

The effects of the COVID-19 pandemic on Latino populations’ PA are likely to have short- and longer-term consequences for health. The findings from this study suggest that social networks among Mexican American immigrants in New York City provided important motivation and support for PA but became harder to sustain during a pandemic, especially given the closure of parks and workplaces and messaging that emphasized staying home and social distancing. The findings can inform strategies to create community environments that are conducive to people maintaining physical activity while at the same time protecting public health.

## Supplementary Information

Below is the link to the electronic supplementary material.

Supplementary file1 (DOCX 14 KB)

## Data Availability

De-identified data from this study will be made available (as allowable according to institutional IRB standards) by emailing the corresponding author.
