# Supplementary material for: Exploring Physical Activity Among Mexican American Immigrants in New York City Before and During the COVID-19 Pandemic: A Two-Wave Panel, Mixed-Method Analysis
Source: J Racial Ethn Health Disparities. 2024 Dec 5;13(1):285–96. doi: 10.1007/s40615-024-02244-1 (PMC12795871; doi:10.1007/s40615-024-02244-1)
Supplement: Supplementary file 1 — Supplementary file1 (DOCX 14 KB) [file 40615_2024_2244_MOESM1_ESM.docx]

**Alternative Table 2**

**Table 2 in terms of odds ratios (for ease of interpretation)**

|  | Adjusted Odds Ratio | 2.5% | 97.5% | P |
| --- | --- | --- | --- | --- |
| Age | 0.856 | 0.699 | 1.019 | **0.095** |
| Female sex | 0.055 | 0.001 | 1.259 | **0.083** |
| Proportion of alters who are “close” | 0.983 | 0.893 | 1.059 | 0.662 |
| Proportion of alters who do PA with participant | 1.058 | 1.008 | 1.122 | **0.032** |
| BMI | 0.929 | 0.767 | 1.120 | 0.427 |
| Marital Status |  |  |  |  |
| Single | REF | REF | REF | REF |
| Married | 0.166 | 0.006 | 3.556 | 0.253 |
| Living with partner | 0.013 | 0.000 | 0.658 | **0.041** |
| Divorced, Separated, or Windowed | 0.808 | 0.018 | 51.273 | 0.913 |
| Working^1^ | 0.987 | 0.074 | 10.274 | 0.991 |
| Number of children <16 yrs old as alters | 0.878 | 0.170 | 4.097 | 0.866 |

Pseudo-R^2^ = 0.92

**Alternative Table 3 (for ease of interpretation)**

**Table 3. Factors associated with change in participants’ IPAQ level from wave 1 to wave 2 (n=49)**

|  | Adjusted Odds Ratio | 2.5% | 97.5% | P |
| --- | --- | --- | --- | --- |
| Baseline IPAQ | 0.017 | 0.002 | 0.083 | **0.000** |
| Age | 1.026 | 0.959 | 1.101 | 0.464 |
| Female sex | 0.228 | 0.034 | 1.182 | 0.095 |
| Change in proportion of alters “close” | 0.936 | 0.878 | 0.989 | **0.026** |
| Change in proportion of alters do PA with | 0.991 | 0.943 | 1.040 | 0.719 |
| BMI | 0.976 | 0.851 | 1.116 | 0.720 |
| Pseudo-R^2^ | 0.74 |  |  |  |
